# Mobile Genetic Elements Driving the Spread of Antimicrobial Resistance in Streptococci

**DOI:** 10.1155/tbed/7984036

**Published:** 2026-10-23

**Authors:** Paulin Mungongo Mayama, Dai Xingyang, Diafuka Saila-Ngita, Wang Liping

**Affiliations:** ^1^ MOE Joint International Research Laboratory of Animal Health and Food Safety College of Veterinary Medicine Nanjing Agricultural University Nanjing 210095 China njau.edu.cn; ^2^ Faculty of Veterinary Medicine University of Kinshasa BP.117 Kinshasa XI Kinshasa Democratic Republic of the Congo unikin.ac.cd; ^3^ Jiangsu Industrial Technology Engineering Center for Risk Prevention and Control of Antimicrobial Resistance in Animal-Origin Bacteria and Development of Innovative Veterinary Drugs Nanjing 210095 China

**Keywords:** antimicrobial resistance, integrative conjugative elements, mobile genetic elements, plasmids, *Streptococcus* spp., zoonotic

## Abstract

Streptococci are adaptable bacteria that persist as commensals or pathogens under selective pressures. This adaptability is influenced by mobile genetic elements (MGEs), which mediate horizontal gene transfer (HGT) and frequently carry antimicrobial resistance (AMR) and virulence‐associated genes. This review provides an overview of MGEs involved in the acquisition and dissemination of AMR across *Streptococcus* species, including human, animal‐associated, and zoonotic streptococci. Integrative and conjugative elements (ICEs), including members of the *Tn916/Tn1545* and *Tn5253* families, and TnGBS‐related ICEs, are among the best‐characterized vehicles for the dissemination of AMR genes (ARGs) because their modular organization supports integration, excision, regulation, and conjugative transfer. Integrative mobilizable elements (IMEs) promote the dissemination of resistance by exploiting conjugative machinery supplied by compatible coresident elements. Plasmids, although less prominent than chromosomally integrated MGEs in several streptococcal species, can carry resistance determinants together with replication and mobilization functions, contributing to HGT. Insertion sequences (ISs) and transposons (Tns) contribute to genome plasticity by promoting gene mobilization, recombination, and the generation of complex resistance regions. Resistance‐associated elements such as *Tn917*, *Tn3872*, *Tn6002*, and *Tn1207.1*‐related structures illustrate the diversity of genetic platforms involved in macrolide and tetracycline resistance. Prophages, including Φm46.1‐like elements, contribute to genome diversification and can serve as determinants of resistance transfer. Genomic islands and cis‐mobilizable elements (CIMEs) expand mobilome diversity through recombination and mobilization in association with compatible ICEs. Collectively, these interconnected MGEs drive the dissemination of AMR, genome plasticity, ecological adaptation, and evolutionary diversification in streptococci. Reducing the need for antimicrobial use in livestock through improved biosecurity, vaccination, hygiene, nutrition, husbandry, disease prevention, and timely diagnosis is therefore essential to reduce selection pressure and limit the persistence and spread of resistance. These measures, integrated with genomic surveillance, antimicrobial stewardship, and One Health approaches, can help limit the circulation of resistance‐associated MGEs across human, animal, and environmental interfaces.

## 1. Introduction

The genus *Streptococcus* comprises a diverse group of Gram‐positive pathogens that cause a broad clinical spectrum in both humans and animals, ranging from superficial mucosal infections to severe invasive conditions such as septicemia, necrotizing fasciitis, and meningitis [1]. Key species of clinical importance include *Streptococcus pyogenes* (Group A Streptococcus [GAS]), *Streptococcus agalactiae* (Group B Streptococcus [GBS]), *Streptococcus pneumoniae*, the pyogenic groups C and G (GCS/GGS), and the *Streptococcus anginosus* group. While GAS is exclusively adapted to the human host [2, 3], causing pharyngitis, pyoderma, and life‐threatening sequelae like rheumatic fever [4] with an estimated global burden of 18 million cases and 517,000 deaths annually [5], GBS colonizes the rectovaginal tract and remains a leading cause of neonatal sepsis and maternal chorioamnionitis [4, 6]. GCS/GGS, historically associated with animal infections [7], comprise several clinically and veterinary‐relevant taxa, including *Streptococcus dysgalactiae* subsp. *equisimilis*, *S. dysgalactiae* subsp. *dysgalactiae*, *S. equi* subsp. *equi*, *S. equi* subsp. *zooepidemicus*, and *S. canis* [8]. Among these, *S. dysgalactiae* subsp. *equisimilis* is an important human pathogen that causes invasive infections, including bacteremia and pneumonia, particularly in vulnerable populations [9–11]. In parallel, the *Streptococcus anginosus* group is implicated with increasing frequency in empyema, endocarditis, and abscesses across various organs [12, 13]. In the veterinary context, zoonotic streptococci such as *S. suis* and *S. parasuis* are major pathogens in swine production, causing meningitis, septicemia, arthritis, and pneumonia, resulting in significant economic losses and zoonotic transmission risks [1, 14, 15]. Although all GAS strains remain highly susceptible to penicillin [16], the escalating crisis of antimicrobial resistance (AMR) severely compromises the efficacy of alternative agents, making management particularly challenging in β‐lactam‐allergic patients across both human and veterinary medicine.

The ongoing AMR crisis has been identified as a major public health challenge for the 21st century [17], with projections estimating up to 10 million annual deaths by 2050 in the absence of effective interventions [18]. The World Health Organization (WHO) has designated macrolide‐resistant GAS, penicillin‐resistant GBS, and macrolide‐resistant *S. pneumoniae* as medium‐priority pathogens on its Bacterial Priority Pathogens List [19], reflecting their substantial contribution to global morbidity and the progressive erosion of treatment options. Surveillance data reveal alarming resistance trajectories: *S. pneumoniae* exhibits widespread reduced susceptibility to penicillin and macrolides, with pooled global rates reaching 57.6% for clarithromycin and 55.8% for azithromycin [20]. In GBS, genomic analyses have documented a fourfold increase in resistance gene abundance over the past 45 years, correlating with phenotypic resistance rates of up to 76.2% for erythromycin, 58.2% for clindamycin, and approximately 89.7% for tetracycline, with Asia emerging as a critical resistance hotspot [20]. Concurrently, GAS macrolide resistance continues to rise, often linked to outbreak‐associated strains [21, 22], and zoonotic streptococci such as *S. suis* and *S. parasuis* display frequent resistance to tetracyclines, macrolides, oxazolidinones, pleuromutilins, and aminoglycosides, driven largely by the antimicrobial use in animal production systems [14, 15].

At the molecular level, the dissemination of AMR genes (ARGs) within streptococcal populations is primarily mediated by horizontal gene transfer (HGT) via mobile genetic elements (MGEs) collectively known as the mobilome [23, 24]. The collection of ARGs, also known as the resistome, is an essential determinant of AMR establishment, further development, and amplification within the microbial community [23]. MGEs, including integrative and conjugative elements (ICEs), transposons (Tns), and plasmids, encode the enzymatic machinery required for intercellular mobility and frequently carry ARGs that confer competitive advantages under antimicrobial selection pressure [25, 26]. Key ARGs identified in streptococci include *erm*(B), *erm*(A), and *mef*(A) for macrolide resistance; *tet*(*M*) and *tet*(O) for tetracycline resistance; *lnu*(B) for lincosamides; *optr*A for phenicol and oxazolidinone resistance; and *lsa*(E) and *lsa*(F) for lincosamide‐streptogramin A‐pleuromutilin resistance [14, 15]. In the animal sector, these MGEs are particularly prevalent in *S. suis* and *S. parasuis* isolates, where they have been identified as primary vectors for the spread of multidrug resistance, reflecting the selective pressure exerted by antimicrobial use in veterinary practice and livestock production [14, 15]. The presence of these MGEs in zoonotic streptococci underscores their role in mediating resistance across human and veterinary sectors, raising concerns about the potential transmission of AMR determinants between animal and human populations [14, 15, 19]. The three classical mechanisms of bacterial HGT are transformation, transduction, and conjugation. Transformation involves the uptake and incorporation of extracellular DNA, transduction involves bacteriophage‐mediated DNA transfer, and conjugation involves transfer mediated by the conjugative machinery. In addition, extracellular vesicle‐associated DNA has emerged as a potential source of genetic material for transformation. In *Streptococcus pneumoniae*, extracellular vesicles carrying surface‐associated bacterial DNA can deliver it to competent recipient cells via the transformation machinery [27–29]. Among these pathways, conjugation mediated by ICEs is particularly significant in streptococci, as these elements often harbor multiple ARGs and virulence determinants, facilitating coselection and the emergence of multidrug‐resistant phenotypes in both human and animal isolates [14, 15, 25, 26]. Understanding the role of MGEs in driving the spread of AMR in streptococci, particularly within the animal sector, where antimicrobial selection pressure is intense and interspecies transmission is a growing concern, is imperative for informing antimicrobial stewardship, refining therapeutic algorithms, and mitigating the expanding threat of multidrug‐resistant streptococcal infections in both human and veterinary medicine. While mutations in pre‐existing genes are a common cause of antibiotic resistance, HGT poses a greater challenge as it is the main mediator of ARGs among bacterial strains and species [29]. The classical mechanisms of HGT are shown in (Figure 1).

**Figure 1:**
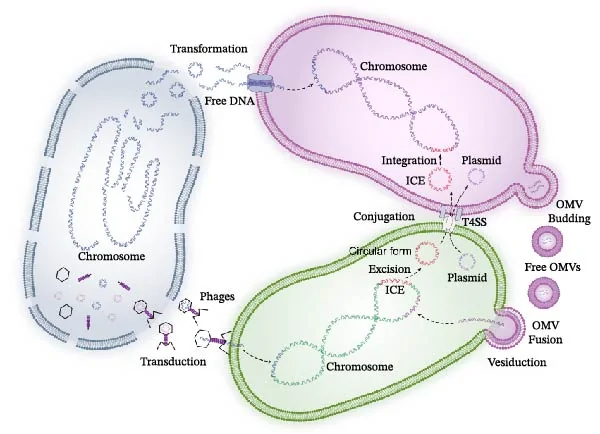
Schematic representation of the major mechanisms of horizontal gene transfer in bacteria. Transformation involves the uptake of extracellular DNA; transduction is bacteriophage‐mediated; and conjugation involves cell‐to‐cell DNA transfer via a conjugative machinery. In addition to these classical mechanisms, extracellular vesicle‐associated DNA transfer has been reported as an emerging route of genetic exchange.

## 2. Classes and Functional Mechanisms of MGEs in Streptococci

HGT is a major driver of AMR dissemination among *Streptococcus* [30, 31]. Resistance genes are transferred primarily through conjugation, although transformation and transduction also contribute to the genetic exchange. This process is largely mediated by MGEs, which facilitate the acquisition, evolution, and spread of ARGs within and between bacterial species. In *Streptococcus* spp., MGEs comprise diverse genetic elements, including plasmids, bacteriophages, Tns, integrons, genomic islands, insertion sequences (ISs), ICEs, and integrative and mobilizable elements (IMEs) [30, 31]. Depending on whether the chromosome‐carried MGEs can self‐transfer through conjugation, ICEs and IMEs have also been defined [30, 32]. Advances in molecular techniques, such as whole‐genome sequencing and CRISPR‐based gene editing, have facilitated a deeper understanding of the genetic architecture of MGEs and their influence on bacterial resistance patterns. Studies have also shown that MGEs are associated with the acquisition of virulence factors, not just resistance genes, thereby enhancing the ability of pathogens such as *Streptococcus pneumoniae* and *Streptococcus pyogenes* to cause severe infections despite antimicrobial treatment [19, 33]. A comprehensive overview of MGEs associated with AMR is provided in (Table S2), and the principal classes of MGEs involved in intracellular mobility and intercellular transfer are summarized in (Figure 2).

**Figure 2:**
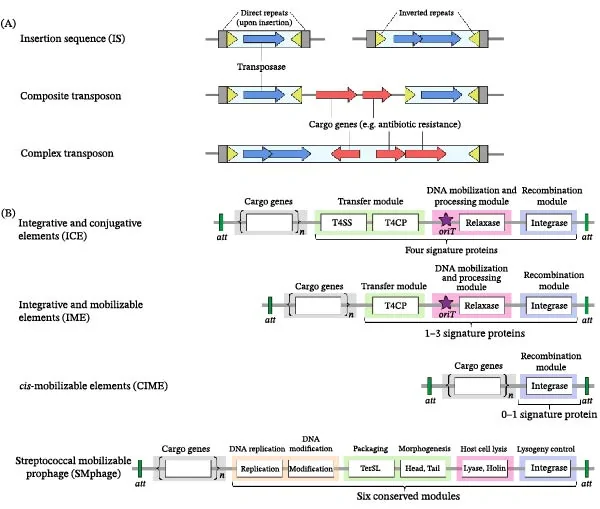
Overview of the principal classes of mobile genetic elements involved in intracellular mobility and intercellular transfer, including (A) insertion sequences and transposons, (B) integrative and conjugative elements, integrative and mobilizable elements, cis‐mobilizable elements, and prophages. CIME, cis‐mobilizable element; ICE, integrative and conjugative element; IME, integrative and mobilizable element; IS, insertion sequence; T4CP, type IV coupling protein; T4SS, type IV secretion system; Tn, transposon.

### 2.1. ICEs

ICEs, also called conjugative Tns, are among the best‐characterized chromosomally integrated MGEs that disseminate AMR across several streptococcal species. ICEs were defined as elements that encode their excision from a host replicon, their intercellular transfer by conjugation, and their integration into a replicon of a recipient cell, regardless of the specificity and mechanism of integration and conjugation [34–36]. Most ICEs encode a tyrosine integrase that catalyzes recombination between identical sequences carried by the *attL* and *attR* recombination sites flanking the element [36, 37]. This enables the excision of a circular form harboring an *attI* site, resulting in a chromosome carrying an *attB* empty site. After transfer, the circular ICE integrates into a replicon, primarily at the 3^′^ end of a tRNA‐ or protein‐encoding gene [38].

Processing of ICE DNA for conjugative transfer is similar to that of conjugative plasmids and analogous to the rolling‐circle replication of some plasmids and viruses [39, 40]. Knowledge of the integration specificity of serine integrases from ICE is scarce. The third family of enzymes, DDE transposases, catalyzes the transposition of DNA segments. The binding of the enzyme to terminal inverted repeats (IRs) at the extremities of elements enables strand cleavage required for the transposition reaction. DDE transposases have low integration specificity and catalyze the duplication of the target sequence (2–13 bp). To date, only one subfamily of DDE transposases has been described in streptococcal ICEs [41, 42]. These DDE integrases catalyze the integration 15–16 bp upstream of the −35 box in the promoter region of various genes.

Studies have confirmed that the ICEs’ integration in streptococcal conferring resistance genes is catalyzed either by a site‐specific tyrosine integrase, a low‐specificity tyrosine integrase, a site‐specific single serine integrase, a triplet of site‐specific serine integrases, or a DDE transposase, and analysis of their integration site led to the detection of target genes for streptococcal ICE insertion, including eight that had not been identified previously, such as (*ftsK*, *guaA*, *lysS*, *mutT*, *rpmG*, *rpsI*, *traG*, *and ebfC*) [34, 41]. *Tn916* is an 18‐kb ICE that belongs to a large family of ICEs, confers tetracycline resistance on its host (via the *tet*M gene), and can transfer to both Gram‐positive and Gram‐negative bacteria. The main families of ICEs carrying ARGs described in *S. suis* are the *Tn916*, *Tn5252*, and *Tn1549* families [43, 44]. In *Streptococcus*, *Tn916*‐like ICEs harbor genes encoding resistance to tetracycline and vancomycin [34, 45, 46]. ICE‐*emm12*, encoding genes for tetracycline and macrolide resistance, was linked to the emergence of scarlet fever *Streptococcus pyogenes* clones in Hong Kong [47, 48]. In *S. pyogenes*, a macrolide‐resistance erm(TR)‐carrying ICE was also identified among outbreak isolates in New Zealand [49]. The acquisition of *tet*(M)‐harboring ICE led to the expansion of tetracycline‐resistant *Streptococcus agalactiae* clones and an increase in neonatal infections [50]. Interestingly, the *tet*(W) tetracycline resistance gene was identified within ICEs in several ruminal bacterial genomes [51]. Recently, the oxazolidinone/phenicol resistance gene *optr*A was identified within an ICESa2603‐family ICE in *Streptococcus suis* [52]. The life cycle of ICEs, including integration, excision, conjugative transfer, and reintegration in recipient cells, is illustrated in (Figure 3).

**Figure 3:**
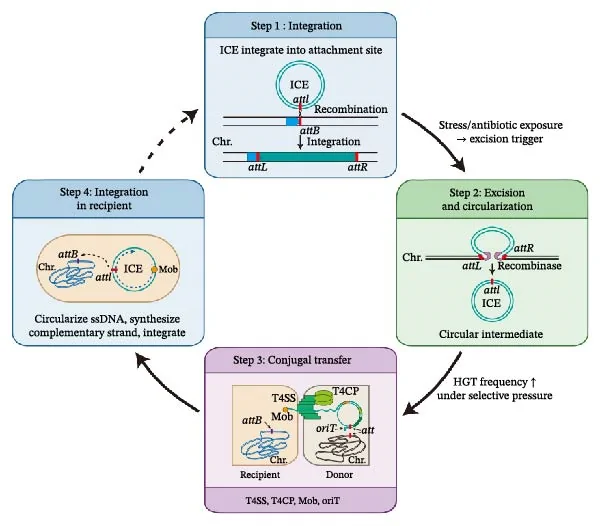
Life cycle of integrative and conjugative elements, showing their integration into the host chromosome, excision and circularization, conjugative transfer to recipient cells, and subsequent reintegration into the genome.

### 2.2. IMEs

The IMEs, also known as mobilizable Tns, are elements that encode their own excision and integration, regardless of mechanism or integration specificity, and can hijack or subvert the mating apparatus of related or unrelated conjugative elements [36]. They are by far the least known elements that transfer by conjugation and are known to encode their own excision and integration machinery and to carry all the sequences or genes necessary to hijack the mating pore of a conjugative element for their transfer. The IMEs from streptococci carry diverse recombination modules and have a large array of integration specificities [53]. Almost all serine integrases from IMEs catalyze site‐specific integration within genes, thereby disrupting them. The majority of IME serine integrases specifically target several conserved genes of the *Tn5252* superfamily, a group of ICEs that is widespread in streptococci [53].

Many IMEs encode a single site‐specific integrase belonging to the tyrosine recombinase family. The tyrosine recombinases recognize the *attR* and *attL* attachment sites flanking the IMEs and catalyze the IME excision by site‐specific recombination that generally takes place between 8 and 60 bp short direct repeats included in the *att* sites [54, 55]. This leads to an extra‐chromosomal circular IME carrying an *attI* site and a chromosome devoid of the IME carrying an *attB* site. After transfer, the recombinase catalyzes integration of the IME via recombination at both the *attI* and *attB* sites on the transconjugant chromosome. As the IME is assumed to replicate by a rolling circle during transfer, it probably reintegrates into the donor cell chromosome after transfer. Most IMEs integrate into specific target sites. Indeed, most IMEs encoding a single tyrosine recombinase integrate in a large array of specific sites, including the 3^′^ end or part of various tRNA genes, the 3^′^ end of genes encoding various housekeeping proteins (such as ribosomal proteins), or their 5^′^ end (more rarely) [55]. Because one of the direct repeats flanking IMEs is at one end of the target gene, their integration does not disrupt it. Some IMEs encoding closely related tyrosine integrases were found to specifically integrate at the conserved nic site of *ori*T in very distantly related ICEs (*Tn916* and ICE*St3*‐related elements) in *S. agalactiae* [56]. Three IMEs found in three different species of *Streptococcus* and encoding related serine integrases are integrated into the same site within *rumA*. This gene encodes a widespread rRNA methyltransferase.

Some *Streptococcus pyogenes* strains resistant to tetracycline and erythromycin harbor an ICE carrying two IMEs [57]. One of them is the *tet*(O) fragment, and the second (IMESp2907) carries the erythromycin resistance gene *erm*(TR). IMESp2907 was also identified (99% DNA identity) in several strains of *S. agalactiae* [58, 59]. Lincosamide resistance genes are also frequent in IMEs. *S. agalactiae*, resistant to lincomycin and susceptible to erythromycin, hosts *tIS*C*pe8*, an IME carrying the *lnu*(P) gene [60]. This element is related to MTn*Sag*1 (IME carrying the *lnu*(C) gene conferring resistance to lincomycin) previously characterized in *S. agalactiae* [61]. Other IMEs (IME_*ori*T) found in *S. agalactiae* carry the *lsa*(C) gene, conferring resistance to lincosamides, streptogramin A, and pleuromutilins [62, 63]. Conjugative elements can mobilize a wide range of nonautonomous genetic elements by providing the transfer machinery required for their intercellular dissemination (Figure 4).

**Figure 4:**
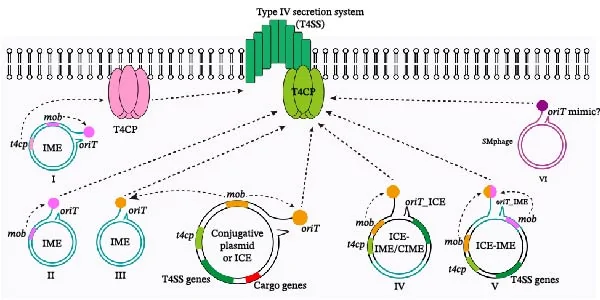
Schematic representation of conjugation‐mediated mobilization of nonautonomous mobile genetic elements, illustrating how conjugative elements provide the transfer machinery required to disseminate IMEs, CIMEs, and prophages between bacterial cells.

### 2.3. Prophage (SMphage Family)

Microbial genomics revealed that in some bacteria, substantial amounts of the bacterial DNA are prophage DNA. Prophages can contribute to bacterial pathogenicity by carrying genes encoding toxins, adhesins, and other virulence‐associated factors, whose expression may modify the phenotype and fitness of the bacterial host [64, 65]. They are reported to harbor ARGs [66–68]. In *Streptococcus*, SMphages harbored ARGs that collectively confer resistance to antimicrobial classes, including the *van*G‐type vancomycin resistance locus and oxazolidinone resistance gene *optr*A. SMphages integrate into four chromosome attachment sites by utilizing three types of integration modules and undergo excision in response to phage induction [68] and four subtypes of Alp‐related surface proteins within SMphages [68]. Certain drug resistance in bacteria is found to be relevant to phages [69], and there are several antibiotic resistance genes carried by bacteriophages, including *tet*A, *tet*W, *int*I1, *int*I2, *int*I3, *bla*OXA‐2, and *bla*TEM [70].

Phage genomes may contain DNA or RNA, and the DNA can be single‐ or double‐stranded. The essential genes of phages include replicase genes, genes encoding proteins involved in DNA packaging, and genes encoding phage components that disrupt the host cell replicative machinery. In *S. mitis*, phage tail genes *pblA* and *pblB* encode adhesion proteins for bacterial host attachment. The process of phage DNA packaging is highly accurate. Still, the host‐genome DNA fragment may be packaged at a finite frequency relative to the phage DNA, resulting in phage particles that can attach to and deliver the DNA to suitable bacteria, with the injected exogenous bacterial DNA integrated into the genome [70].

### 2.4. Complex Transposon (Unit Tns)

Unit Tns were traditionally thought of as elements larger than the IS, bounded by IRs rather than by a pair of ISs, and including a transposase gene and an internal “passenger” gene(s) that may encode antibiotic resistance. This IS/Tn distinction is becoming more problematic; however, as there are now examples of relatives of well‐known IS‐carrying passengers (transporter IS [*tIS*]) [71], which may have a Tn name/number, and cryptic relatives of Tns without any passengers, which may be given an IS name. The transposon registry [72] lists and provides counts of unit Tns, and some *Tn3*‐family Tns are included in the ISfinder [73]. Antibiotic resistance genes are often associated with *Tn3-family Tns*, *as* reviewed by Nicolas et al. [74]. Another transposon superfamily, referred to here as *Tn7*‐like Tns, includes members associated with antibiotic resistance, such as *Tn7*‐ and *Tn402*‐like elements in Gram‐negative bacteria [74]. The *Tn3* family and *Tn7*‐like complex Tns most relevant to antibiotic resistance in streptococci are associated with the *Tn916 tet*(M) gene, *Tn1545* carrying resistance genes *erm*(B), *tet*(O), *Tn3872* resistance to macrolides *erm*(B), *Tn6000* carrying *tet*(M), *Tn917 erm*(A), *Tn5393 cat*A, and *Tn1207.1*carrying *aac*(3)IV gene [10, 74].

### 2.5. Plasmids

Plasmids are extrachromosomal DNA molecules that can auto‐replicate and are distributed to daughter cells when the bacterium divides within a suitable host. Plasmids can transfer wild‐type high‐frequency resistance and virulence determinants from one bacterium to another (even of another species) via HGT [75]. However, plasmids are generally less prominent than chromosomally integrated MGEs in several streptococcal species; however, their abundance and contribution vary substantially among species and lineages. Resistance‐associated plasmids have been characterized in *S. agalactiae*, *S. pyogenes*, *S. suis*, *S. parasuis*, and several other streptococci [10, 76]. The transfer of chromosomal DNA may be affected by plasmid transfer during HGT due to plasmids’ ability to mobilize chromosomes [77].

Plasmids have been grouped by replicon, and the streptococcal plasmid pMV158 belongs to the Rep_2 family (PF01719). These examples illustrate that plasmids can play a role in the spread of resistance in certain contexts: pIP501 confers resistance to macrolides by methylating the 23S rRNA *erm*(B) and through an efflux mechanism *mef*(A) in *Streptococcus agalactiae* (GBS), pSLA2 provides resistance to penicillin *bla*(Z) and contributing to macrolide resistance in some strains *erm*(B) in *Streptococcus pneumoniae*, pG+: *Tn6000* confers resistance to tetracycline *tet*(M) and to macrolides *erm*(A) various *Streptococcus* species (including *Streptococcus pyogenes*), pRUM resistance to tetracycline *tet*(M) in *Streptococcus mutans* and pS1 resistance to aminoglycosides *aac*(3)‐IV in *Streptococcus suis* [10, 76, 78, 79]. Diverse plasmids harboring ARGs in *Streptococcus* spp. are summarized in (Table S1).

### 2.6. Transposable Elements: ISs and Composite Tns

Transposable elements are mobile DNA sequences capable of changing the genomic location through transposase‐ or recombinase‐mediated mechanisms. In bacteria, their movement may occur within a chromosome or between replicons, including chromosomes and plasmids. When transposable elements are embedded within conjugative plasmids, ICEs, or other transferable MGEs, they may also disseminate between bacterial cells through HGT. Following insertion, these elements can be maintained through clonal inheritance and contribute to the genome rearrangement, acquisition of resistance genes, and bacterial adaptation [31, 32]. Transposable elements were first discovered by the American geneticist McClintock in 1951 [2] and were widely recognized in 1967 when Shapiro identified them in *Escherichia coli*. Initially, transposable elements were considered unusual, but they were later regarded as selfish DNA capable of replicating autonomously [10]. Evidence subsequently demonstrated that Tns play important roles in genome evolution [10, 80]. TEs are commonly found in chromosomes, ICEs, bacteriophages, and plasmids.

In prokaryotes, transposable elements can be classified into two main categories: ISs and Tns [10]. ISs are among the simplest bacterial transposable elements and generally encode a transposase flanked by terminal IRs. Composite Tns arise when two IS elements flank additional genetic material, including ARGs, allowing the intervening region to mobilize as a single unit. A compound or composite transposon was originally defined as a segment of DNA located between two copies of the same IS, in which the entire structure moves as a single discrete unit composed of both IS elements and the central DNA segment [81].

These transposable elements are key facilitators of bacterial evolution and adaptation and contribute to the emergence and dissemination of antibiotic and heavy‐metal resistance, virulence determinants, and other pathogenic traits. Composite Tns, in particular, contribute to the assembly and rearrangement of multidrug‐resistance regions in bacterial genomes.

In streptococci, several resistance‐associated transposable and composite elements have been characterized. *Tn917* carries the macrolide‐lincosamide‐streptogramin B resistance determinant *erm*(B), whereas *Tn3872* is a composite structure resulting from the insertion of *Tn917* into a *Tn916*‐like element, thereby linking *erm*(B) with the tetracycline resistance gene *tet*(M). *Tn6002* is another *Tn916*‐related element carrying both *erm*(B) and *tet*(M) and has been identified in *Streptococcus pneumoniae*, *S. pyogenes*, and other streptococci. *Tn916* and *Tn1545* are more appropriately classified as conjugative Tns or ICEs rather than conventional composite Tns. *Tn916* principally carries *tet*(M), whereas *Tn1545* carries *tet*(M), *erm*(B), and *aphA-3*, conferring resistance to tetracyclines, macrolide–lincosamide–streptogramin B antibiotics, and aminoglycosides, respectively [82].

Several other IS elements have also been associated with one or more antibiotic‐resistance genes within transposon‐like structures in streptococci [83]. These include IS256, associated with various antibiotic resistance genes, including determinants for macrolide and tetracycline resistance; IS30, involved in the mobilization of antibiotic‐resistance genes, particularly in *Streptococcus pneumoniae*; IS1216, found in *Streptococcus agalactiae* and associated with resistance genes; IS1485, identified in *Streptococcus pneumoniae* and potentially involved in the transfer of resistance genes; and IS431 in *Streptococcus pyogenes*. These IS elements are known to participate in the assembly of multiple resistance‐associated structures that resemble composite Tns [84].

### 2.7. Cis‐Mobilizable Element (CIME)

CIMEs are non‐autonomous chromosomal mobile elements that lack the complete recombination and conjugation machinery required for independent transfer but retain cis‐acting sequences that can be recognized by the machinery of a related ICE. When paired with a compatible ICE, CIMEs can exploit ICE‐encoded recombination and conjugation functions for excision, transfer, and chromosomal integration. Experimental studies in streptococci have demonstrated ICE‐mediated cis‐mobilization of genomic islands, thereby establishing CIMEs as a distinct class of nonautonomous MGEs [85, 86].

In *Streptococcus suis*, resistance‐associated CIMEs have also been identified within ICE‐associated regions. Examples include CIMESsu14ND71, CIMESsu14ND96, and CIMESsu14ND2, which contain AMR determinants and illustrate CIMEs’ contribution to the organization and dissemination of multidrug resistance regions. These elements may contribute to both the transmission of ARGs and the structural evolution of streptococcal ICEs [87, 88].

## 3. Comparative Distribution and Transmission Network of Major MGEs in Streptococci

The distribution of MGEs among *Streptococcus* species is heterogeneous and influenced by bacterial ecology, genomic compatibility, evolutionary history, and antimicrobial selection pressure. Different MGEs show preferential associations with specific species or lineages, contributing to the acquisition and spread of AMR and adaptive traits. Their dissemination mainly occurs through HGT, particularly conjugation mediated by ICEs, IMEs, plasmids, and Tns, while transformation and transduction provide additional transfer routes. Recombination and environmental selection further promote the persistence and diversification of MGE‐associated resistance determinants. The distribution of major MGEs among important streptococci is summarized in (Table 1).

**Table 1:** Comparative distribution and main contribution of mobile genetic elements (MGEs) among major streptococci.

Species	ICEs/IMEs	Transposons	Plasmids	Prophages/phage‐related elements	Main contribution of MGEs	References
*Streptococcus pneumoniae*	Frequent	Frequent	Occasional	Frequent	AMR acquisition and genome adaptation	[2, 10, 34, 44, 69, 88–90]
*Streptococcus pyogenes*	Present	Present	Rare to occasional	Very frequent	Virulence evolution and phage‐mediated adaptation	[10, 45, 48, 82, 91]
*Streptococcus agalactiae*	Frequent	Frequent	Occasional	Frequent	AMR spread and host adaptation	[10, 50, 57, 59, 60, 62, 63, 86]
*Streptococcus mutans*	Present	Present	Occasional	Present	Oral niche adaptation and biofilm fitness	[10, 34, 55, 92]
*Streptococcus suis*	Very frequent	Frequent	Present	Present	Multidrug‐resistance dissemination and extensive horizontal gene transfer	[14, 15, 52, 53, 82, 93–95]
*Streptococcus parasuis*	Present–frequent	Present	Present	Present/poorly characterized	Emerging AMR reservoir and genomic diversification	[96, 97]
*Streptococcus thermophilus*	Present	Limited reports	Occasional	Frequent	Phage‐driven evolution and industrial adaptation	[10, 35, 55, 85]
Other oral streptococci (*S. mitis*, *S. oralis*, *S. salivarius*, *S. sanguinis*)	Frequent	Present	Occasional	Present	Reservoir of accessory genes and horizontal gene exchange	[55, 56, 88, 92]
*Streptococcus equi* subsp. *equi*	Present	Limited reports	Limited reports	Present	Genome diversification and horizontal gene transfer	[10, 34, 55]
*Streptococcus equi* subsp. *zooepidemicus*	Present	Limited reports	Limited reports	Present	Genome diversification and horizontal gene transfer	[10, 34, 55]
*Streptococcus dysgalactiae* subsp. *dysgalactiae*	Present	Present	Limited reports	Present	AMR dissemination and genome plasticity	[10, 34, 55]
*Streptococcus dysgalactiae* subsp. *equisimilis*	Present	Present	Limited reports	Present	AMR dissemination and horizontal gene exchange	[8–10, 34, 55]

### 3.1. Host Range and Genomic Distribution of MGEs in Streptococci

MGEs display distinct host ranges and genomic distribution patterns across the genus *Streptococcus*, reflecting their adaptation to particular bacterial lineages and ecological niches. Comparative genomic analyses reveal that ICEs are the most broadly disseminated MGEs among streptococci of clinical and veterinary importance, including *Streptococcus pneumoniae*, *S. pyogenes*, *S. agalactiae*, *S. suis*, and *S. parasuis*. Members of the *Tn*916 and *Tn*5252/*Tn*5253 families have been identified across multiple species, suggesting sustained horizontal transfer within the genus [30, 96]. In contrast, prophages exhibit restricted distribution; their diversity and abundance vary considerably both between species and among closely related strains [68]. *S. pyogenes* harbors one of the most prophage‐enriched genomes within the genus, whereas the mobilomes of *S. pneumoniae* and *S. suis* contain numerous ICEs. In *S. suis*, ARG‐associated prophage‐ or prophage‐related elements include ΦSC181, ΦSsu1135/10, ΦSsUD.1, and ΦJH1301. In contrast, Φm46.1‐like elements carrying resistance determinants have been described in both *S. pyogenes* and *S. suis*. Plasmids are less prevalent than chromosomally integrated MGEs but have been consistently reported in *S. agalactiae*, *S. suis*, and, more recently, *S. parasuis*, where they contribute to genome diversification and genetic plasticity [14, 30, 96].

The genomic distribution of MGEs across the genus *Streptococcus* is highly non‐random. ICE integration is largely determined by the sequence specificity of their cognate integrases, which recognize compatible chromosomal attachment sites (*attB*) [30, 34]. These sites may occur within conserved tRNA genes or housekeeping loci, where specific sequences provide stable targets for site‐specific recombination [34, 55, 62]. Integration at the 3^′^ end of such genes may minimize disruption of essential host functions because recombination can preserve or reconstruct the functional target sequence [62]. Prophages and other integrated MGEs similarly use defined chromosomal attachment sites via integrase‐mediated site‐specific recombination; in streptococci, the conserved *rum*A locus is a documented integration site for several MGEs [14, 55, 82, 89]. In contrast, plasmids are maintained as extrachromosomal replicons and exhibit substantial diversity in size and genetic content [10, 59]. Comparative genomic analyses further demonstrate that individual streptococcal genomes may harbor multiple MGEs simultaneously, generating mosaic architectures through successive integration, excision, recombination, and acquisition events [14, 15, 89]. Several MGE integration sites are conserved among streptococci, whereas the composition and diversity of the mobilome vary substantially among species and lineages, emphasizing its dynamic contribution to genome evolution [14, 15, 89, 92].

### 3.2. Contribution of MGEs to Genome Plasticity, Adaptation, and Evolution

Beyond the dissemination of AMR, MGEs are major contributors to the genome plasticity and adaptive evolution in streptococci. Their recurrent acquisition, loss, excision, integration, and recombination modify accessory gene content and generate lineage‐specific genome architectures [35, 98]. ICEs and IMEs can introduce resistance, metabolic, bacteriocin‐associated, and other accessory functions, while recombination between related elements can generate new mosaic structures with altered host ranges or cargo genes. In *Streptococcus agalactiae*, ICEs and related elements contribute substantially to the genome diversity [57], whereas in *S. suis*, chromosomal conjugative and mobilizable elements carry diverse resistance‐ and bacteriocin‐associated genes and constitute an important component of the accessory genome [53]. Recombination between streptococcal ICEs can also generate hybrid elements, as demonstrated by the formation of an ICE derived from *S. suis* ICESsu32457 and *S. agalactiae* ICESa2603 that was transferable to *S. pyogenes* [99].

Prophages provide an additional mechanism of evolutionary diversification by introducing accessory genes and promoting genome rearrangement and lysogenic conversion [82, 83]. In *S. pneumoniae*, mobilome‐rich genomes containing multiple integrated elements illustrate how successive MGE acquisition contributes to genome diversification. Similarly, genomic analyses of oral streptococci have revealed an extensive diversity of chromosomally integrated MGEs, supporting their contribution to adaptation within complex microbial communities [89]. In *S. salivarius*, the abundance and diversity of ICEs and IMEs have been directly associated with environmental adaptation [100].

### 3.3. Ecological Drivers of MGEs Selection and Persistence in Streptococci

The persistence and dissemination of MGEs in streptococci are influenced by ecological, evolutionary, and anthropogenic factors operating across the human–animal–environment interface. Antimicrobial exposure represents an important selective pressure favoring the maintenance of resistance‐associated MGEs. In human‐associated streptococci, *Tn*916/*Tn*1545‐family ICEs carrying resistance determinants such as *tet* (*M*) and, in some derivatives, *erm*(*B*) are widely distributed and transferable among oral streptococci [2, 44, 45]. In animal‐associated streptococci, particularly *S. suis*, ICEs and IMEs serve as important reservoirs and vehicles for ARGs [14, 53]. The physical linkage of multiple resistance determinants within the same MGE can additionally promote coselection under antimicrobial pressure [14, 96].

Additionally, subinhibitory antimicrobial concentrations, frequently detected in wastewater and agricultural effluents, stimulate conjugation and activate SOS responses, which facilitate HGT. Host‐associated microbial ecosystems further promote the maintenance and persistence of MGEs. The oral mucosal niche, respiratory tract, and gastrointestinal tract constitute highly dynamic microbial habitats with polymicrobial biofilms, where commensal streptococci, including *S. mitis*, *S. oralis*, and *S. mutans*, function as genetic reservoirs, facilitating the exchange of resistance determinants with pathogenic streptococcal lineages through conjugation and natural transformation within dental plaque and mucosal surfaces. Similar dynamics occur in animal hosts, where *Streptococcus suis* in swine, *S. uberis* and *S. agalactiae* in dairy cattle, and *S. equi* in horses harbor MGEs encoding both resistance‐ and virulence‐associated traits [68]. Frequent ICE‐mediated genetic exchange among cocolonizing bacterial lineages within tonsillar and mammary niches further contributes to the persistence and dissemination of these mobile elements. Biofilms represent particularly permissive ecological niches for MGE persistence and dissemination. The extracellular polymeric matrix enhances bacterial cell‐to‐cell contact, promotes conjugative transfer, facilitates competence development, and provides protection against antimicrobial stress [68].

Furthermore, biofilm‐associated microbial interactions can mitigate the fitness costs imposed by MGE acquisition through metabolic cooperation and community‐level adaptation, allowing MGEs to persist even in the absence of direct antimicrobial selection. Evolution further contributes to the long‐term stabilization of these elements as adaptive mutations occur either within the bacterial chromosome or in MGE carriage, thereby explaining the sustained prevalence of resistance‐associated MGEs despite antimicrobial stewardship interventions. Many streptococcal MGEs also carry genetic determinants associated with resistance to heavy metals (copper and zinc), biocides, and oxidative stress [68]. In intensive livestock production systems, dietary metal supplementation and extensive disinfectant use generate persistent coselective pressures that sustain the maintenance and dissemination of multidrug‐resistant MGEs independently of direct antimicrobial exposure. These production systems represent major evolutionary hotspots, where high stocking densities, frequent antimicrobial interventions, and intensive management practices facilitate the emergence and diversification of novel MGE architecture across swine, dairy cattle, and poultry populations.

Growing genomic evidence demonstrates that MGEs connect ecological compartments through direct animal contact, food production systems, environmental contamination, and wastewater networks, thereby facilitating the widespread circulation of resistance‐associated genetic material between human and animal microbiomes. Environmental reservoirs, including manure, agricultural soils, sediments, and surface waters, act as persistent repositories of MGE diversity, enabling recurrent cycles of gene mobilization, environmental persistence, and subsequent reintroduction into host‐associated microbial communities [68]. These interconnected processes, including antimicrobial selection, biofilm‐mediated horizontal transfer, compensatory evolution, nonantibiotic coselection, intensive animal production, cross‐host dissemination, and environmental recycling, collectively shape the remarkable stability, adaptability, and ecological persistence of the streptococcal mobilome.

## 4. Experimental and Computational Approaches in MGEs Research

Research on MGEs in streptococci increasingly relies on integrated experimental and computational approaches to characterize their structure, mobility, and biological significance. Experimentally, the activity and transfer potential of MGEs are commonly assessed through PCR detection of excision or circular intermediates, conjugation, and transformation assays, and, when necessary, mutational analyses of key transfer genes. These approaches have been used to demonstrate the prevalence and transferability of *Tn916/Tn1545*‐family iICEs in oral streptococci and to show that streptococcal mobilizable prophages can disseminate via ICE‐assisted conjugation, thereby expanding the routes for the spread of resistance and virulence determinants [68, 96].

Complementing these laboratory methods, computational genomics has become essential for identifying and comparing MGEs across streptococcal populations. Whole‐genome sequencing, particularly hybrid long‐read and short‐read assemblies, enables the accurate reconstruction of complex elements such as nested Tns, ISs, and composite islands. Comparative genomic analyses have revealed extensive diversity and broad distribution of conjugative and chromosomally integrated MGEs across multiple streptococcal species [101, 102]. In addition, specialized tools such as ICEscreen, PHASTER, ISEScan, and MGEfinder facilitate the detection of ICEs, IMEs, prophages, and ISs, although no single pipeline is sufficient for complete mobilome reconstruction. Therefore, the most reliable studies combine automated prediction with manual curation and experimental validation to distinguish intact mobile elements from degraded remnants and to link genomic data to biological function [90, 98, 103, 104]. A summary of current research approaches and emerging tools used in the study of MGEs is provided in (Table S3).

## 5. Future Perspectives and Control Strategies

As evidence continues to accumulate that MGEs are major drivers of AMR and adaptive evolution in streptococci, future research must move beyond descriptive genomics toward actionable control strategies. This shift requires both mechanistic interventions that directly limit HGT and surveillance systems capable of detecting emerging resistance trajectories across human, animal, and environmental reservoirs. In this context, the integration of molecular tools, genomic epidemiology, and One Health surveillance frameworks offers a promising route for reducing the circulation of high‐risk MGEs and the genes they carry [68, 93, 105].

### 5.1. Targeted Interventions to Curb HGT

Targeted suppression of HGT represents a key future direction in limiting the spread of resistance‐associated MGEs in streptococci. One promising approach is the use of CRISPR‐Cas‐based antimicrobials to selectively eliminate resistance genes or cure mobile elements from bacterial populations. Because many AMR determinants are carried on conjugative or mobilizable elements, sequence‐specific CRISPR strategies could reduce the persistence of these elements without broadly disrupting the microbiota [68, 93]. In parallel, interventions that interfere with excision, conjugation, or prophage mobilization may reduce the transfer efficiency of ICEs, IMEs, and prophage‐associated elements. This is particularly relevant in streptococci, where ICE‐assisted conjugative dissemination of prophages has already been demonstrated, emphasizing that multiple classes of MGEs may cooperate during transmission.

In addition, reducing antimicrobial use in livestock should be regarded as an important complementary strategy for limiting the selection, persistence, and dissemination of resistance‐associated MGEs. Preventive measures, including enhanced biosecurity, vaccination, improved hygiene, appropriate stocking densities, optimized nutrition, and good animal husbandry practices, can reduce the incidence of infectious diseases and, consequently, the need for antimicrobial treatment. Early disease detection, accurate veterinary diagnosis, and targeted antimicrobial therapy can further reduce unnecessary or empirical antimicrobial use. Antimicrobial stewardship is therefore essential as reducing antimicrobial exposure and selection pressure may reduce the fitness advantage conferred by resistance determinants and limit the persistence of resistance‐carrying MGEs within bacterial populations [68, 93]. In streptococci, decreased antimicrobial selection pressure may consequently reduce the maintenance and dissemination of ARG‐bearing ICEs, IMEs, plasmids, Tns, and other MGEs, thereby limiting their horizontal transfer within livestock‐associated bacterial communities. Thus, strategies aimed at reducing antimicrobial demand should complement direct interventions targeting MGE transmission and contribute to a broader approach for controlling the emergence and spread of AMR.

### 5.2. Predictive Monitoring Under the One Health Framework

Effective long‐term control of MGEs‐mediated resistance also depends on predictive monitoring systems grounded in the One Health concept. Because AMR emerges and circulates across interconnected human, animal, and environmental interfaces, surveillance must extend beyond clinical isolates to include livestock, wildlife, food systems, wastewater, and other environmental reservoirs. Genomic surveillance is particularly valuable in this regard as it enables simultaneous tracking of bacterial lineages, resistance genes, and the MGEs that mediate their movement [68, 105]. Such approaches can identify high‐risk clones, detect newly mobilized resistance determinants, and reveal transmission pathways that would be missed by phenotype‐based monitoring alone. Future surveillance efforts should therefore integrate whole‐genome sequencing, standardized meta‐data collection, and cross‐sector data sharing to support early warning systems and more accurate risk prediction. Among streptococci, this type of integrated monitoring will be especially important for identifying emerging MGE networks linking commensal reservoirs to clinically important pathogens [68, 105]. As illustrated in (Figure 5), AMR in *Streptococcus* circulates across the human–animal–environment interface through interconnected One Health pathways, driven by inappropriate antibiotic use and multiple transmission routes.

**Figure 5:**
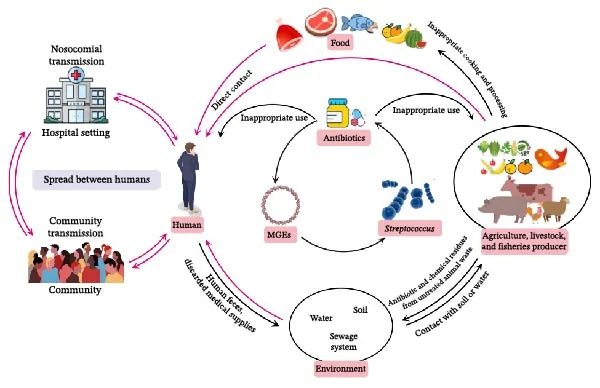
One Health pathways of antimicrobial resistance in *Streptococcus*, showing transmission among humans, animals, food, and the environment, driven by inappropriate antibiotic use and spread via direct contact, contaminated food, and environmental exposure.

## 6. Conclusion and Remarks

Streptococci possess a highly dynamic mobilome that contributes substantially to the acquisition, maintenance, and dissemination of AMR determinants. ICEs, IMEs, transposable elements, plasmids, prophages, genomic islands, and CIMEs collectively facilitate HGT among streptococcal lineages and, in some cases, between different bacterial species. Among these elements, *Tn916/Tn1545*‐ and *Tn5252/Tn5253*‐related ICEs are particularly well characterized and illustrate how modular genetic structures can combine integration, excision, conjugative transfer, and AMR functions [1, 10, 14, 34, 54]. Plasmids and prophage‐associated elements provide additional routes for the dissemination of resistance determinants, whereas IMEs and CIMEs exploit conjugative functions supplied by compatible mobile elements [53, 82, 85, 86].

Importantly, the biological significance of MGEs extends beyond AMR. Recurrent acquisition, loss, integration, excision, and recombination of MGEs contribute to genome plasticity, accessory gene variation, and the emergence of mosaic genomic structures. ICEs and related elements contribute substantially to the genome diversity in *S. agalactiae*, while the extensive repertoire of conjugative and mobilizable elements in *S. suis* underscores their roles in both the dissemination of AMR and genome diversification [14, 53, 57, 106]. Recombination between related ICEs can also generate hybrid elements with altered genetic composition and transfer capacity, demonstrating the evolutionary connectivity of streptococcal mobilomes [91]. Prophages further contribute to genome diversification through lysogenic conversion, recombination, and the acquisition of accessory genes [82, 83]. *S. suis* represents a particularly well‐characterized example of this dynamic process, harboring diverse ICEs, IMEs, prophage‐associated elements, and resistance determinants [14, 52, 54, 94, 95, 97, 107]. The 89 K‐associated region found in epidemic *S. suis* lineages further illustrates how integrated mobile regions can combine conjugative functions with determinants that contribute to bacterial adaptation and pathogenicity [94, 104, 105]. Comparable processes have been documented across human–animal‐associated, zoonotic, and commensal streptococci, indicating that MGE‐mediated evolution is a genus‐wide phenomenon rather than one confined to individual species.

Overall, the streptococcal mobilome should be regarded not only as a reservoir and vehicle of ARGs but also as an important driver of genome plasticity, ecological adaptation, and evolutionary diversification. The capacity of streptococci to acquire and exchange resistance genes through MGEs, including between commensal and pathogenic strains, represents an important challenge for both human and animal health and may compromise the long‐term effectiveness of newly developed antimicrobials. Continued genomic surveillance, experimental characterization of MGE transfer, antimicrobial stewardship, and integrated One Health approaches are therefore essential for detecting emerging resistance‐associated genes and limiting their circulation across human–animal and environmental interfaces.

## Author Contributions

**Paulin Mungongo Mayama**: conceptualization, investigation, formal analysis, writing – original draft, writing – review and editing. **Dai Xingyang**: investigation, formal analysis, writing – review and editing. **Diafuka Saila-Ngita**: writing – review and editing. **Wang Liping**: conceptualization, supervision, funding acquisition, writing – review and editing.

## Funding

This study was supported by the National Natural Science Foundation of China (Grant 32473094), the National Key Research and Development Program of China (Grant 2022YFD1800400), Fundamental Research Funds for the Central Universities (Grant KYLH2025013), and the Priority Academic Program Development of Jiangsu Higher Education Institutions.

## Ethics Statement

The authors have nothing to report.

## Conflicts of Interest

The authors declare no conflicts of interest.

## Supporting Information

Additional supporting information can be found online in the Supporting Information section.

## Supporting information


**Supporting Information** Additional supporting information is available online in the Supporting Information section. It includes Table S1: ARG‐associated plasmids in *Streptococcus* species. Table S2: Mobile genetic elements (MGEs) associated with antimicrobial resistance in *Streptococcus* species. Table S3: Current research approaches and emerging tools in MGE studies.

## Data Availability

No new data were generated or analyzed in support of this review. All the data cited in this article are available in the referenced publications. Data sharing does not apply to this article.
