# Supplementary material for: Mobile Genetic Elements Driving the Spread of Antimicrobial Resistance in Streptococci
Source: Transbound Emerg Dis. 2026 Oct 23;2026:7984036. doi: 10.1155/tbed/7984036 (PMC13693407; doi:10.1155/tbed/7984036)
Supplement: Supplementary file 1 [file TBED-2026-7984036-s001.docx]

**Supplementary**

**Table S1. ARGs-associated plasmids in *Streptococcus* *spp*.**

| Host | Strain | Plasmid | Acc. No. | Antimicrobial resistance genes |
| --- | --- | --- | --- | --- |
| *Streptococcus agalactiae* | BSU452 | pip501 | KP698941 | *aac(6')-aph(2'')* |
| *Streptococcus agalactiae* | NGBS128 | pNGBS128 | CP012742 | erm(T) |
| *Streptococcus agalactiae* | CCH208 | pCCH208 | NC_024973 | erm(T) |
| *Streptococcus agalactiae* | GB2001 | pGB2001 | NC_015973 | erm(T) |
| *Streptococcus agalactiae* | GB2002 | pGB2002 | NC_015971 | erm(T) |
| *Streptococcus agalactiae* |  | pGB354 | NC_001797 | cat(pC221) |
| *Streptococcus agalactiae* |  | pIP501 | NC_002136 | erm(B) |
| *Streptococcus agalactiae* |  | pLS1 | NC_001380 | tet(L) |
| *Streptococcus agalactiae* |  | pMV158 | NC_010096 | tet(L) |
| *Streptococcus alactolyticus* |  | - | NZ_PV575690 | lsa(E), lnu(B), ant(6)-Ia, spw |
| *Streptococcus alactolyticus* |  | - | NZ_PV575695 | tet(O/W/32/O), tet(L), lnu(A), Δerm(B) |
| *Streptococcus alactolyticus* |  | - | NZ_PV575699 | tet(O/W/32/O), tet(L), lnu(A), Δerm(B) |
| *Streptococcus alactolyticus* |  | - | NZ_PV575724 | lsa(E), lnu(B), ant(6)-Ia, spw |
| *Streptococcus dysgalactiae* | V305 | pSdyV305 | OQ299016 | fexA, poxtA, cfr(D) |
| *Streptococcus dysgalactiae subsp. equisimilis* | Sde5580 | p5580 | NC_019370 | erm(T) |
| *Streptococcus gallolyticus* |  |  | MZ503509 | cfr(D) |
| *Streptococcus gallolyticus subsp. gallolyticus* | ATCC BAA-2069 | pSGG1 | NC_015219 | tet(O/W/32/O), tet(L) |
| *Streptococcus infantis* |  | pSI01 | NC_019365 | tet(S) |
| *Streptococcus iniae* | FP5228 |  | CP024844 | erm(B), cat |
| *Streptococcus mutans* | MD |  | CP044494 | tet(C), erm(B), catA1 |
| *Streptococcus parasuis* | H35 | pH35-cfrD | CP076722 | cfr(D) |
| *Streptococcus parauberis* | AD112410 | pAD112410 | CP195078 | tet(S) |
| *Streptococcus parauberis* | HFTC0064 | pSPseroIa | CP104191 | tet(S) |
| *Streptococcus parauberis* | KRS02083 |  | CP082784 | tet(S) |
| *Streptococcus parauberis* | SPOF21J26 | pSPseroIa | CP119429 | tet(S) |
| *Streptococcus parauberis* | SPOF3K |  | CP025421 | tet(S), tet(M) |
| *Streptococcus pasteurianus* | AL101002 | pAL101002 | KU511280 | erm(T), tet(M), tet(L) |
| *Streptococcus pasteurianus* | WUSP082 |  | CP136944 | optrA, fexA, erm(A) |
| *Streptococcus pluranimalium* | SS-15 |  | CP161816 | lnu(A), lsa(F) |
| *Streptococcus pluranimalium* | XJSP | pXJSP-1 | NZ_JBQZYV010000062 | lnu(A) |
| *Streptococcus pneumoniae* | R6 | pMV158 | MZ396393 | tet(L) |
| *Streptococcus pneumoniae* |  |  | AY452662 | bla_TEM-129_ |
| *Streptococcus pyogenes* | FDAARGOS_514 |  | CP033816 | erm(C) |
| *Streptococcus pyogenes* | FDAARGOS_668 |  | CP044094 | erm(C) |
| *Streptococcus pyogenes* | GA2000 | pGA2000 | NC_019252 | erm(T) |
| *Streptococcus pyogenes* | Spyo01 |  | CP136949 | erm(T) |
| *Streptococcus pyogenes* | Spyo09 |  | CP136952 | erm(T) |
| *Streptococcus pyogenes* | TSPY556 | pRW35-like | CP032701 | erm(T) |
| *Streptococcus pyogenes* |  | pBT233 | NG_047797 | erm(B) |
| *Streptococcus pyogenes* |  | pDB101 | NC_006978 | erm(B) |
| *Streptococcus pyogenes* |  | pMD101 | X66468 | erm(B) |
| *Streptococcus pyogenes* | 9116-03 | pRW35 | NC_010423.2 | erm(T) |
| *Streptococcus pyogenes* |  | pSM19035 | NC_006979 | erm(B) |
| *Streptococcus raffinosi* | LM8 |  | NZ_JBOZIT010000018 | Δtet(C) |
| *Streptococcus salivarius* | ICDC2 |  | CP018188 | lnu(C) |
| *Streptococcus suis* | 3366 | unnamed2 | KX785327 | tet(O) |
| *Streptococcus suis* | 3370 | unnamed1 | KX785329 | tet(O/W/32/O) |
| *Streptococcus suis* | 3372 | unnamed2 | KX785323 | erm(B) |
| *Streptococcus suis* | 34391 | unnamed2 | CP185346 | tet(O) |
| *Streptococcus suis* | D06037420 | - | FJ411062 | tet(B) |
| *Streptococcus suis* | D06038342 | - | FJ411063 | tet(B) |
| *Streptococcus suis* | D06038425 | - | FJ411064 | tet(B) |
| *Streptococcus suis* | D06040055 | - | FJ411066 | tet(B) |
| *Streptococcus suis* | D06040301 | - | FJ411065 | tet(B) |
| *Streptococcus suis* | D06060811 | - | FJ411061 | tet(B) |
| *Streptococcus suis* | D08003974 | - | FJ411076 | tet(B) |
| *Streptococcus suis* | D08004897 | - | FJ411069 | tet(B) |
| *Streptococcus suis* | D08005424 | - | FJ411071 | tet(B) |
| *Streptococcus suis* | D08005499 | - | FJ416863 | tet(B) |
| *Streptococcus suis* | D08005838 | - | FJ411073 | tet(B) |
| *Streptococcus suis* | D08005902 | - | FJ411067 | tet(B) |
| *Streptococcus suis* | D08006055 | - | FJ411070 | tet(B) |
| *Streptococcus suis* | D08006583 | - | FJ411074 | tet(B) |
| *Streptococcus suis* | D08006584 | - | FJ411072 | tet(B) |
| *Streptococcus suis* | D08006802 | - | FJ411068 | tet(B) |
| *Streptococcus suis* | D08006872 | - | FJ411075 | tet(B) |
| *Streptococcus suis* | DNC13 | pD13_3 | CP102151 | erm(B) |
| *Streptococcus suis* | DNR43 | pD43_2 | CP102144 | erm(B) |
| *Streptococcus suis* | DNR48 | pD48_2 | CP102142 | erm(B) |
| *Streptococcus suis* | DNS20 | pD13_3 | CP102147 | erm(B) |
| *Streptococcus suis* | ID24665 | pID24665SS2 | CP135067 | erm(B) |
| *Streptococcus suis* | S10 | pStrcfr | KF129409, KC844836 | cfr, lnu(E), fexA |
| *Streptococcus suis* | SC262 | pSC262 | CP061278 | erm(T) |
| *Streptococcus suis* | SH0505 |  | HQ113109 | tet(L) |
| *Streptococcus suis* | SH0918 | pSH0918 | MT180124 | *optrA* |
| *Streptococcus suis* | SS2401 | pSS2401.2 | NZ_JBQWEH010000003 | aph(3')-III, erm(B) |
| *Streptococcus suis* | Ssuis_MA4 |  | CP084909 | tet(O) |
| *Streptococcus suis* | T15 | pT15_2 | CP100433 | *vanZ* |

**Table S 2. Major types of MGEs associated with antimicrobial resistance in Streptococcus *spp***

| MGEs | Hosts | AMR genes | Integration site (s) | Integrase family | References |
| --- | --- | --- | --- | --- | --- |
| ICEs |  |  |  |  |  |
| Tn*916* | *S. suis*, *S. agalactiae, S. pneumoniae*  *S. mutants* | *tet*(M) | *llmO* | Tyrosine | [53] |
| *dICE_Tn916* | *S. suis* | *tet*(O)  *tet*(M) | - | - | [53] |
| *Tn5252*  *ICE_Tn5252_rplL* | *S. suis, S. agalactiae, S. pneumoniae, S. pyogenes* | *tet*(O), *tet*(O/W/32/O*), tet*(40*), erm*(B*), aph*(3’)- *IIIa, ant*(9’), *ant*(6’)-*Ia, sat*4 | *rpl*L | Tyrosine | [53] |
| *ICE_Tn5252_rumA* | *S. suis, S. agalactiae, S. pneumoniae, S. pyogenes* | *tet*(M), *tet*(L), *tet*(O*), tet*(O/W/32/O), *erm*(B), *aph*(3’)-*III, ant*(6’)*-Ia, sat*4 | *rum*A | Tyrosine | [14, 52, 53, 90] |
| *ICE_Tn5252_mutT* | *S. suis* | *tet*(L), *tet*(O), *tet*(O/ W/32/O*), erm*(B), *msr*(C), *mef*(A) | *mut*T | Tyrosine | [14, 53] |
| *ICE_Tn5252_rbgA* | *S. suis* | *erm*(B) | *rbg*A | Tyrosine | [53] |
| *ICE_Tn5252_ llmO* | *S. suis* | *tet*(M) | *llm*O | Tyrosine | [53] |
| *dICE_Tn5252* | *S. suis* | *tet(O), tet(40)* | *rpl*L | Tyrosine | [53] |
| *ICESpsSUT-286-1* | *S. parasuis* | *tet*(M), *ant*(6)-*Ia* | *-* | - | [108] |
| *ICESpsH35–1* | *S. parasuis* | *msr*(D), *erm*(B*)* | *-* | - | [108] |
| *ICESps89–4109-1-1* | *S.parasuis* | *erm*(B), *tet*(O) | *-* | - | -[108] |
| *ICESpsSUT-319-1* | *S. parasuis* | *erm*(B), *ant*(6)-*Ia* | *-* | - | [108] |
| *ICESpsSUT-328-1* | *S. parasuis* | *erm*(B), *ant*(6)-*Ia* | *-* | - | [108] |
| Partial_Tn5252 | S. suis | *tet*(O/W/32/O), *erm*(B), *aph*(3’)-IIIa, ant(9’), *sat*4 | *rplL* | Tyrosine | [53] |
| ICE*Ss*D9 | *S. suis* | *tet*(O), *erm*(B) | *rplL* | Tyrosine | [90, 93] |
| ICE*Ssu*32457 | *S. suis, S. agalactiae, S. pneumoniae, S. pyogenes* | *tet*(40), *tet*(O/W/32/O), *erm*(B), *ant*(6’)-Ia*, aph*(3’) | *rplL* | Tyrosine | [90, 106, 109] |
| ICE*Ssu*SC216 | *S. suis* | *tet*(O), *erm*(B), *optrA, aadD* | *rplL* | Tyrosine | [52] |
| Tandem_ICE*Ssu*SC317  ICE*Ssu*BSB6 | *S. suis*  *S. suis* | *tet*(L), *tet*(O), *optrA*  *tet*(O/W/32/O), *erm*(B), *aph*(3’)-IIIa*, ant*(6’)-Ia*, sat4, vanG* operon | *rum*A  *rplL* | SR  Tyrosine | [52]  [102] |
| ICE*Ssu*JH1308-2 | *S. suis* | *tet*(M), *ant*(6’)-Ia | *rplL* | Tyrosine | [14] |
| ICE*Ssu*JH1301 | *S. suis* | *tet*(O), *erm*(B), *aph*(3’)-III | *rplL* | Tyrosine | [14] |
| Tn1549  dICE_Tn*1549_rplL* ICE_Tn*1549_rbgA*  Tn*1549*  ICE*Sp*1108 | *S. suis*  *S. suis*  *S. suis*  *S. suis,*  *S. agalactiae,*  *S. pneumoniae,*  *S. pyogenes* | *tet*(O/W/32/O) *erm*(B)  *vanB* operon  *tet*(L), *tet*(W), *erm*(B), *lnu*(B), *erm* (TR), *ant* (6’)-Ia, *cat* | *rplL*  *rbgA*  *hsM*  *rumA* | Tyrosine  Tyrosine  Tyrosine  Tyrosine | [53]  [53]  [14] |
| *VanG*  ICE_vanG_lysS | *S. suis* | *tet*(W) | *lysS* | - | [53] |
| Tn*GBS2*  ICE_Tn*GBS2*  ICE*SsuTYPE3_rplL* | *S. suis*, *S. agalactiae*  *S. suis, S. agalactiae, S. pneumoniae, S. pyogenes* | tet(L), *aph*(3’)-IIIa, *ant*(6)  *tet*(L), *tet*(W), *erm*(B), *lnu*(B), *ant*(6’)-Ia*, cat* | Diverse insertion sites, but not *rplL*  *rplL* | Tyrosine  Tyrosine | [110]  [14] |
| IMEs  Integrated in MGEs  IME_*PPI* | *S. suis, S. thermophilus* | *tet*(O), *tet*(40), *erm*(B) | *PPI* | SR | [53] |
| IME_*SNF2* | *S. suis* | *tet*(O), *erm*(B) | *SNF2* | SR | [53] |
| Integrated in chromosome IME_*Ssu*NSUI181_*tRNALeu* | *S. suis* | *aph*(3’)-IIIa, *ant*(6)-Ia, *sat4, vanZ* | *tRNA-leu* | Tyrosine | [53] |
| IME_*Ssu*NSUI231_*rpsI* | *S. suis* | *tet*(O) | *rpsI* | Tyrosine | [53] |
| IME_HTH-XRE-reg | *S. suis* | *ant*(6)-Ia | HTH-XRE regulator | SR | [53] |
| IME_*CJB111_rpsI* | *S. agalactiae* |  |  | Tyrosine | [57] |
| IME*Ssu*YS111 | *S. suis* | *AR, erm*(B), *AR, tet*(O) | *snf2* |  | [88] |
| IME_*GB00957_oriT* | *S. agalactiae* |  |  |  | [62, 111] |
| IME_*SpnAP200_rumA* | *S. Pneumoniae* |  | *in rumA* |  | [55, 111] |
| IME_*SpnG54_rumA* | *S. Pneumoniae* |  | *in rumA* |  | [55] |
| IME_*SpnHun19A_tRNAleu* | *S. Pneumoniae* |  | 3'tRNA^leu^ long_DR |  | [55] |
| IME_*Ssal*1001175st1_H3_oriT | *S. salivarius* | *qacE, dfrA10, qacE, sul1* | 3' *rpmG* adjacent |  | [55, 94] |
| IME*Sp2907* | *S. pyogenes* |  | Internal site of *orf8 from ICESp2905 and orf10 from ICESp1108* |  | [94] |
| Prophages Prophage_*rumA* | *S. suis* | *tet*(O/W/32/O), *tet*(W), *erm*(B), *lnu*(B), *lnu*(C), *lsa*(E), *mef*(A), *ant*(6’)-Ib, *aph*(3’)-IIIa, *ant*(9’), *sat4* | *rumA* | - |  |
| *ΦSC181* | *S. suis* | *mef*(A), *aacA, aphD, cat, optrA* | *rumA* |  | [52] |
| *Φm46.1* | *S. suis*, *S. pyogenes* | *tet*(O), *mef*(A) | *rumA* | Site-specific recom-binase | [14, 52] |
| *ΦSsu1135/10* | *S. suis* | *erm*(B), *ant*(6’)-Ia, *aph*(3)-IIIa | *rumA* | - | [107] |
| *ΦSsUD.1* | *S. suis*, *S. pyogene*s | *tet*(W), *erm*(B), *ant*(6’)-Ia, *aph*(3’)-III*, sat4* | *rumA* | - | [52, 107] |
| *ΦJH1301* | *S. suis* | *mef*(A), *ant*(6’)-Ia*, cat, sat4* | *cysM / rumA /* SSU1958 | - | [14] |
| CIME  CIME*Ssu*14ND71 | *S. suis* | *erm(B), aph(3')-III, aac(6')-aph(2''), tet(O/W/32/O)* | *snf2* |  | [88] |
| CIME*Ssu*14ND96 | *S. suis* | *erm*(B), *aph*(3')-III, *aac*(6')-*aph*(2''), *tet*(O/W/32/O) | *snf2* |  | [88] |
| CIME*Ssu*14ND2 | *S. suis* | *erm*(B), *aph*(3')-III, *aac*(6')-*aph*(2''), *tet*(O/W/32/O) | *snf2* |  | [88] |

**Table S 3. Current research approaches and emerging tools for MGE studies**

| Approach/tool | Main purpose | Typical output | Major strengths | Main limitations |
| --- | --- | --- | --- | --- |
| WGS (short-read Illumina) | Detect MGE genes | Draft genomes | Accurate, scalable | Poor repeat resolution |
| Long-read sequencing | Resolve full MGEs | Complete elements | Good structural detail | Higher error rate |
| Hybrid assembly | Build full mobilomes | High-quality assemblies | High accuracy | Costly, complex |
| Comparative genomics/pangenome | Compare MGE diversity | Gene presence patterns | Population-scale insight | Depends on assembly quality |
| Conjugation / filter-mating assays | Test transfer ability | Transfer frequency | Functional proof | Limited to lab conditions |
| Transformation assays | Measure DNA uptake | Transformation rate | Useful in competent strains | Host-dependent |
| PCR/qPCR / ddPCR excision assays | Detect excision events | Copy number/excision rate | Sensitive, targeted | Not genome-wide |
| Phage induction assays | Test prophage activation | Phage release data | Reveals prophage activity | Not always inducible |
| Gene deletion/complementation | Validate MGE genes | Functional evidence | Mechanistic insight | Labor-intensive |
| ICEfinder / ICEberg 3.0 | Predict ICEs / IMEs | Candidate regions | ICE-focused | Needs manual checking |
| ICEscreen | Detect ICEs / IMEs | Annotated elements | Good for Firmicutes | Less robust on fragmented genomes |
| PHASTER | Identify prophages | Prophage regions | Easy prophage annotation | May overpredict remnants |
| ISEScan | Detect insertion sequences | IS annotation | Automated IS calling | Sensitive to assembly quality |
| MOB-suite | Reconstruct plasmids | Plasmid types/contigs | Good for plasmid analysis | Limited for complex plasmids |
| Metagenomics | Survey community MGEs | Resistome/mobilome | Community-level view | Difficult host assignment |
| Machine learning/network analysis | Predict spread patterns | Risk scores/networks | Predictive value | Needs validation |
